# High Hepatitis B Prevalence and Vaccination Needs Among Transgender Women and Men Sex Workers in Barcelona, Spain

**DOI:** 10.1093/ofid/ofae410

**Published:** 2024-07-17

**Authors:** Adrián Antuori, Anna Not, Jocelyn Mesías-Gazmuri, Victoria González, Marcos Montoro-Fernandez, Cinta Folch, Verónica Saludes, Marta Villar, Mercè Meroño, Sonia Paytubi, Laia Alemany, Jordi Casabona, Elisa Martró, SexCohort Group, C Folch, L Ferrer, V González, R Muñoz, J Mesías-Gazmuri, L Egea, J Casabona, E Martró, V Saludes, A Antuori, A Not, S González-Gómez, L Alemany, M A Pavón, S Paytubi, A Esteban, M Vergara, V Rodríguez, F Morey, S Tous, A Montoliu, S de Sanjosé, L Villegas, M Villar, H Adell, K Santander, M Meroño, M Cebrián, C Carrascal, E Longares, L Martínez, R Almirall, R Mansilla, P Lletjós, G Drou-Roget, A Álvarez-Vargas

**Affiliations:** Microbiology Department, Laboratori Clínic Metropolitana Nord (LCMN), Hospital Universitari Germans Trias i Pujol, Institut d’Investigació Germans Trias i Pujol (IGTP), Badalona (Barcelona), Spain; Genetics and Microbiology Department, Universitat Autònoma de Barcelona (UAB), Bellaterra (Barcelona), Spain; Microbiology Department, Laboratori Clínic Metropolitana Nord (LCMN), Hospital Universitari Germans Trias i Pujol, Institut d’Investigació Germans Trias i Pujol (IGTP), Badalona (Barcelona), Spain; Genetics and Microbiology Department, Universitat Autònoma de Barcelona (UAB), Bellaterra (Barcelona), Spain; Centre d’Estudis Epidemiològics sobre Infeccions de Transmissió Sexual i Sida de Catalunya (CEEISCAT), Agència de Salut Pública de Catalunya, Barcelona, Spain; Germans Trias i Pujol Research Institute (IGTP), Campus Can Ruti, Badalona, Spain; Department of Pediatrics, Obstetrics and Gynecology and Preventive Medicine, Universitat Autònoma de Barcelona (UAB), Bellaterra (Barcelona), Spain (Jocelyn, Jordi); Microbiology Department, Laboratori Clínic Metropolitana Nord (LCMN), Hospital Universitari Germans Trias i Pujol, Institut d’Investigació Germans Trias i Pujol (IGTP), Badalona (Barcelona), Spain; Centre d’Estudis Epidemiològics sobre Infeccions de Transmissió Sexual i Sida de Catalunya (CEEISCAT), Agència de Salut Pública de Catalunya, Barcelona, Spain; Consorcio de Investigación Biomédica en Red de Epidemiología y Salud Pública (CIBERESP), Instituto de Salud Carlos III (ISCIII), Madrid, Spain; Centre d’Estudis Epidemiològics sobre Infeccions de Transmissió Sexual i Sida de Catalunya (CEEISCAT), Agència de Salut Pública de Catalunya, Barcelona, Spain; Centre d’Estudis Epidemiològics sobre Infeccions de Transmissió Sexual i Sida de Catalunya (CEEISCAT), Agència de Salut Pública de Catalunya, Barcelona, Spain; Germans Trias i Pujol Research Institute (IGTP), Campus Can Ruti, Badalona, Spain; Consorcio de Investigación Biomédica en Red de Epidemiología y Salud Pública (CIBERESP), Instituto de Salud Carlos III (ISCIII), Madrid, Spain; Microbiology Department, Laboratori Clínic Metropolitana Nord (LCMN), Hospital Universitari Germans Trias i Pujol, Institut d’Investigació Germans Trias i Pujol (IGTP), Badalona (Barcelona), Spain; Consorcio de Investigación Biomédica en Red de Epidemiología y Salud Pública (CIBERESP), Instituto de Salud Carlos III (ISCIII), Madrid, Spain; STOP, Barcelona, Spain; Fundació Àmbit Prevenció, Barcelona, Spain; Cancer Epidemiology Research Programme, Catalan Institute of Oncology, IDIBELL, L’Hospitalet de Llobregat, Barcelona, Spain; Consortium for Biomedical Research in Epidemiology and Public Health—CIBERESP, Carlos III Institute of Health, Madrid, Spain; Cancer Epidemiology Research Programme, Catalan Institute of Oncology, IDIBELL, L’Hospitalet de Llobregat, Barcelona, Spain; Consortium for Biomedical Research in Epidemiology and Public Health—CIBERESP, Carlos III Institute of Health, Madrid, Spain; Centre d’Estudis Epidemiològics sobre Infeccions de Transmissió Sexual i Sida de Catalunya (CEEISCAT), Agència de Salut Pública de Catalunya, Barcelona, Spain; Germans Trias i Pujol Research Institute (IGTP), Campus Can Ruti, Badalona, Spain; Department of Pediatrics, Obstetrics and Gynecology and Preventive Medicine, Universitat Autònoma de Barcelona (UAB), Bellaterra (Barcelona), Spain (Jocelyn, Jordi); Consorcio de Investigación Biomédica en Red de Epidemiología y Salud Pública (CIBERESP), Instituto de Salud Carlos III (ISCIII), Madrid, Spain; Microbiology Department, Laboratori Clínic Metropolitana Nord (LCMN), Hospital Universitari Germans Trias i Pujol, Institut d’Investigació Germans Trias i Pujol (IGTP), Badalona (Barcelona), Spain; Consorcio de Investigación Biomédica en Red de Epidemiología y Salud Pública (CIBERESP), Instituto de Salud Carlos III (ISCIII), Madrid, Spain

**Keywords:** hepatitis B virus, men sex workers, risk factors, trans women sex workers, vaccination

## Abstract

**Background:**

Transgender women sex workers (TWSWs) and men sex workers (MSWs) are especially vulnerable to acquiring hepatitis B virus (HBV) infection. We aimed to describe HBV prevalence (hepatitis B surface antigen [HBsAg] and core antibody [HBcAb]) and associated risk factors for HBV exposure (HBcAb), to assess vaccination status and risk factors for no prior vaccination, and to compare HBV prevalence and vaccination status between TWSWs and MSWs.

**Methods:**

The SexCohort study was advertised to TWSWs and MSWs through several communication channels. At cohort entry through 2 community-based organizations in Barcelona, the study population was screened for HBV and other sexually transmitted infections, and an epidemiological questionnaire was administered (n = 271).

**Results:**

Overall, 93.0% of participants were migrants, mostly from South and Central American countries. HBsAg prevalence was 1.9% (TWSWs, 2.4%; vs MSWs, 0.9%; *P* = .42), and previous exposure to HBV was 31.8% (TWSWs, 38.5%; vs MSWs, 20.8%; *P* = .002). Over 5 years of sex work (adjusted odds ratio [aOR], 9.35), prior exposure to *Treponema pallidum* (aOR, 3.49), and treatment with anxiolytic drugs (aOR, 3.23) were associated with HBV exposure. Overall, 33.7% of participants exhibited immunity from vaccination (TWSWs, 30.8%; vs MSWs, 38.61%; *P* < .001), while 34.4% were candidates to HBV vaccination (TWSWs, 30.8%; vs MSWs, 40.6%; *P* < .001). Never having been on pre-exposure prophylaxis for HIV (odds ratio [OR], 4.23) and non-Spanish origin (OR, 5.00) were associated with no prior HBV vaccination.

**Conclusions:**

There is a need to reinforce screening and vaccination programs aimed at TWSWs and MSWs as integrated services offered at the community centers commonly accessed by these populations.

Globally in 2019, an estimated 316 million people had chronic hepatitis B virus (HBV) infection, the leading cause of death from liver cancer and the third cause of death from cirrhosis [1, 2]. In 2016, the World Health Assembly adopted the first Global Health Sector Strategy to eliminate viral hepatitis as a public health threat. This initiative underscored the importance of equitable access to interventions, like screening and HBV vaccination, focusing especially on key risk populations such as people who inject drugs, men who have sex with men (MSM), migrants, people in prison, and sex workers (SWs) [3]. Although efforts have been made in recent decades to eliminate hepatitis B (mainly aimed at preventing mother-to-child transmission and transfusion safety), innovative approaches are needed to reach diagnosis and treatment rate targets set by the World Health Organization (WHO) elimination strategy.

In Spain, the prevalence of HBV surface antigen (HBsAg) is 0.22% in the general population [4]. However, SWs are vulnerable to sexually transmitted infections (STIs) including HBV infection, due to several factors, including exposure to many sexual partners, risky sexual practices, and drug use [5]. The prevalent marginalization, violence, and criminalization they encounter exacerbate this vulnerability [5]. Therefore, prioritizing vaccination programs [6, 7] and regular HBV testing [8] within integrated screening services for infectious diseases like HIV and other STIs is imperative [9]. However, few studies address HBV risk factors and vaccination specifically in SWs, more so in the subgroups of transgender women sex workers (TWSWs) [10] and cisgender men sex workers (MSWs) [11]. These populations, additionally characterized by a high proportion of migrants, may experience several types of barriers including stigma when accessing health care services [12] and frequently access community-based centers offering rapid testing and social services.

In a previous study conducted among TWSWs and MSWs accessing community-based centers in Barcelona (the Sweetie project), a high prevalence of STIs, including HBV exposure in 34.2%, was uncovered, with 69% of participants being unaware of their status [13]. The present study aimed to (i) characterize the prevalence of HBV active infection and resolved infection and associated risk factors for HBV exposure; (ii) assess vaccination status and risk factors for no prior vaccination; and (iii) compare HBV prevalence and vaccination status between TWSWs and MSWs accessing community-based organizations (CBOs) in Barcelona in the context of the SexCohort project offering screening of bacterial and viral STIs.

## METHODS

### Study Design

The SexCohort Project was a prospective, open-label cohort of TWSWs and MSWs recruited in 2 community-based centers in Barcelona that offered free, anonymous, voluntary, and confidential counseling and testing for viral hepatitis, HIV, and other STIs, aiming to describe their prevalence and determinants [14]. The sample size was calculated based on the estimated population size of MSWs and TWSWs in Barcelona, which is ∼350 individuals. This estimation considered both those accessing community centers and those not linked to any center. In addition, it was taken into account that 14% of this population would decline to participate in the study, resulting in a target recruitment of 300 participants.

### Study Participants and Recruitment

Recruitment, from May 2019 to July 2021, was carried out through convenience sampling, by referral from the network of contacts and acquaintances of participating individuals who attended community centers in Barcelona, in order to invite other individuals who met the inclusion criteria. Additionally, informative materials, including an information sheet and an explanatory brochure, were distributed through other community-based centers in Barcelona. Advertising was also conducted on sector websites such as Pasión, Telechapero, Sexchapero, and HUNQZ.

Inclusion criteria were being of legal age, having worked as a sex worker in the previous 3 months (defined as people who exchange sex, regularly or occasionally, for money), living in Spain (including those participants with a permit and those without it, such as those with pending permit applications or irregular administrative situations), signing the informed consent form, and agreeing to return for follow-up visits. They were compensated €10 and answered an anonymous survey, administered by trained staff, encompassing sociodemographic details, sex work specifics, drug and alcohol use, discrimination experienced in different community settings, self-reported testing, previous diagnosis and treatment of viral hepatitis and HIV/STIs, HBV vaccination, and pre-exposure prophylaxis (PrEP) for HIV. Questions related to discrimination were asked with an adapted scale used in previous studies in transgender population [15].

Participants were given unique identification codes linking biological samples with their surveys. The study was conducted according to the principles of the Declaration of Helsinki and was approved by the Ethics Board at Hospital Universitari Germans Trias i Pujol, Badalona. Details about the study and characteristics of participants have been previously described [16].

### Biological Sample Collection and STI Testing

A venepuncture blood sample was collected to test for HBV, hepatitis C virus (HCV), HIV, and syphilis. Urine, pharyngeal, and anal samples were collected to test for *Neisseria gonorrhoeae* (NG) and *Chlamydia trachomatis* (CT).

NG and CT DNA was detected by real-time polymerase chain reaction using the BD MAX CT/GC assay (Beckton Dickinson). *Treponema pallidum* antibodies (TPAs) were detected using the VITROS Chemistry System (Ortho Clinical Diagnostics); positive results were confirmed by *T. pallidum* hemagglutination (TPHA; Bio-Rad Laboratories). Anti-HCV antibodies were detected using the VITROS system, and if positive, HCV viral load was quantified with the Abbott RealTime HCV assay (Abbot Molecular). HBsAg, anticore antibody (total HBcAb), and surface antibody (HBsAb) were detected using the VITROS system. For participants testing positive for either HBsAg or HBcAb in the absence of HBsAb (“isolated HBcAb positivity”), HBV viral load was quantified using the Abbott RealTime HBV assay (Abbott Molecular). For HIV, a rapid diagnostic test was used (Determine HIV—1/2, Abbott), and if reactive, HIV viral load was quantified from plasma with the Abbott RealTi*me* HIV-1 assay (Abbott Molecular). Those individuals testing positive for any of these STI tests were referred to the health care system for further testing and clinical assessment.

Regarding HBV serological status, participants were categorized according to the Centers for Disease Control and Prevention (CDC) classification [8]: HBV infection (HBsAg-positive), immunity due to natural HBV infection (HBcAb-positive and HBsAb-positive), immunity due to vaccination (positive only for HBsAb, although this marker may be negative due to nonresponse in 5%–15% of vaccinated persons [17]), vaccination candidates (susceptible; negative for all serological markers), and participants who were only positive for HBcAb (isolated HBcAb positivity). For analysis purposes, all those participants with HBV infection, immunity due to natural HBV infection, or isolated HBcAb positivity were classified as previously exposed to HBV.

### Data Analysis

First, we performed a descriptive analysis of sociodemographic characteristics, behavioral profile, and previous viral hepatitis/HIV/STI diagnosis and treatment. We incorporated variables for syphilis, NG, and CT detection from any specimen (urine, pharyngeal, and anal samples).

Second, we conducted logistic regression models to estimate crude and adjusted odds ratios (aORs) along with their respective 95% CIs to evaluate risk factors of HBV exposure. Participants classified as previously exposed to HBV were compared with those susceptible to HBV infection (negative for all HBV serological markers). Participants with serological evidence of vaccination (HBsAb-positive and HBcAb-negative) were excluded from this analysis. Variables deemed potential risk factors from the existing literature were incorporated; those with a *P* value <.10 in the unadjusted regression model were included in the adjusted analysis.

Finally, we conducted an analysis to investigate risk factors for no prior vaccination. We considered 2 groups: participants with serological evidence of vaccination (HBsAb-positive and HBcAb-negative) and participants susceptible to HBV infection (negative for all HBV serological markers). Participants with previous exposure to HBV were excluded from this analysis. The chi-square and Mann-Whitney *U* test were used to compare proportions, and we set a cutoff for statistical significance at *P* < .05. A logistic regression model to estimate crude odds ratios along with their respective 95% CIs was used. Missing values were not included in the analysis. We used R 4.3.1 “Beagle Scouts” for the analyses.

## RESULTS

### Study Population

The main characteristics of the 271 participants recruited are shown in Table 1. Among them, 62.7% were TWSWs and 37.3% were MSWs. The overall median age was 41 years, and 56.1% were above 30 years of age (TWSWs, 65.9%; and MSWs, 39.6%; *P* < .001). Ninety-three percent of participants were migrants from countries with low or intermediate HBV prevalence, primarily from South and Central America (86.0%), and 44.5% had Spanish residency. Over half of the participants (53.1%) had engaged in sex work for >5 years (TWSWs, 68.2%; and MSWs, 27.7%; *P* < .001).

**Table 1.** Sociodemographic and Bio-behavioral Profile of Participants, and Differences Among Transgender Women Sex Workers and Male Sex Workers

	TWSWs (n = 170)		MSWs (n = 101)		Total (n = 271)		*P* Value
	No.	%/SD	No.	%/SD	No.	%/SD	
Sociodemographic characteristics							
Age >30 y	112	65.9	40	39.6	152	56.1	<.001
Non-Spanish origin	160	94.1	92	91.1	252	93.0	.485
Residency permission	82	48.8	36	37.1	118	44.5	.086
Primary education or less	51	30.0	16	15.8	67	24.5	.014
Bio-behavioral characteristics							
>5 y of sex work	116	68.2	28	27.7	144	53.1	<.001
Age at first sexual relation, y	13.49	3.4	14.33	3.0	13.80	3.3	.036
Condomless sex in the last 12 mo	78	47.0	80	81.6	158	59.9	<.001
No. of clients in the last wk	7.94	8.6	5.09	6.2	6.87	7.9	.002
Stable partner	73	42.9	62	61.4	135	49.8	.005
Alcohol consumption in the last 12 mo (before or during sex)	116	69.1	66	65.4	182	67.7	.621
Drug consumption in the last 12 mo	131	78.9	82	82.0	213	80.1	.652
Drug injection (ever in life)	0	0.0	7	6.9	7	2.6	.001
Drug snorting (ever in life)	136	81.0	82	81.2	218	81.0	1.000
Chemsex in the last 12 mo	35	21.0	39	38.6	74	27.6	.003
Ever been in prison	25	14.9	9	8.9	34	12.6	.216
Diagnosed of STI in the last 12 mo	39	22.9	32	31.7	71	26.2	.150
Hepatitis B vaccination (self-reported)	…	…	…		…	…	.671
No	36	21.4	20	20.0	56	20.9	
I don't know	68	40.5	46	46.0	114	42.5	
Yes	64	38.1	34	34.0	98	26.8	
HIV PrEP treatment (ever in life)	6	3.6	12	11.9	18	6.7	.017
Experienced discrimination in medical facilities (ever in life)	53	31.9	16	16.2	69	26.0	.005
Physical aggression or sexual abuse in the last 12 mo	45	26.8	20	19.8	65	24.2	.251
Anxiolytic treatment in the last 12 mo	52	31.0	21	20.8	73	27.1	.094
Antidepressant treatment in the last 12 mo	26	15.6	11	10.9	37	13.8	.372

Abbreviations: MSWs, male sex workers; OR, odds ratio; PrEP, pre-exposure prophylaxis; STI, sexually transmitted infection; TWSWs, transgender women sex workers.

Furthermore, 59.9% of participants reported condomless sex in the previous 12 months, 27.6% engaged in chemsex (the deliberate use of drugs or substances to enhance sexual experiences over an extended duration) in the previous 12 months, and 2.6% had injected drugs ever in life; 12.6% had been in prison at some point in their lives, 26.2% had been diagnosed with an STI in the previous 12 months, and 6.7% had received PrEP treatment at some point (TWSWs, 3.6%; and MSWs, 11.9%; *P* = .017). Overall, 26.0% had experienced discrimination in a medical facility ever in life (31.9% of TWSWs and 16.2% of MSWs; *P* = .005), 24.2% had faced physical aggression or sexual abuse in the previous 12 months, and 27.1% of participants had been treated with anxiolytics.

### HBV Infection Prevalence and Risk Factors for HBV Exposure

Of the 271 participants recruited, 1 was excluded due to lack of clinical samples for testing. HBsAg prevalence was 1.9% (5/270; 95% CI, 0.6%–4.3%), 2 being new diagnoses (Table 2). Of the 5 participants with HBV infection (HBsAg-positive), all were coinfected with HIV (none of them with HCV). Additionally, 4 had been diagnosed with an STI in the previous 12 months.

**Table 2.** HBV Serological Status and Differences Among Transgender Women Sex Workers and Male Sex Workers

	TWSWs (n = 169)		MSWs (n = 101)		Total (n = 270)		*P* Value
HBV Serological Status^a^	No.	%	No.	%	No.	%	
HBV infection	4	2.37	1	0.99	5	1.85	.42
Immunity due to natural HBV infection	47	27.81	14	13.86	61	22.59	<.001
Immunity due to vaccination	52	30.77	39	38.61	91	33.70	<.001
Susceptible (vaccination candidate)	52	30.77	41	40.59	93	34.44	<.001
Isolated HBcAb positivity^b^	14	8.28	6	5.94	20	7.40	<.001

Abbreviations: HBV, hepatitis B virus; MSWs, male sex workers; TWSWs, transgender women sex workers.

^a^CDC classification (2023). HBV infection: HBsAg-positive, HBcAb-positive. Immunity due to resolved natural HBV infection: HBsAg-negative, HBcAb-positive, HBsAb-positive. Immunity due to vaccination: HBsAg-negative, HBcAb-negative, HBsAb-positive. Susceptible, never infected (vaccination candidate): HBsAg-negative, HBcAb-negative, HBsAb-negative.

^b^Can be the result of a past infection when HBsAb levels have waned, occult infection, a false positive (susceptible), or a mutant HBsAg strain that is not detectable by laboratory assay (infection).

When focusing on HBV exposure, a 31.9% (86/270; 95% CI, 26.3%–37.8%) prevalence of HBcAb was observed. Participants with HBV exposure were mainly from 4 South American countries: Colombia (24.4%), Peru (12.8%), Ecuador (12.8%), and Venezuela (12.8%). Regarding immunity due to natural resolved infection (both HBcAb- and HBsAb-positive in the absence of HBsAg), 22.6% (61/270) participants exhibited this serological profile (Table 2). Among these, 43.0% were HIV-positive and 72.13% had been diagnosed with an STI in the previous 12 months. Additionally, 7.4% (20/270) of participants were only positive for HBcAb (isolated HBcAb positivity) (Table 2).

In the adjusted model, >5 years of sexual work (aOR, 9.35; 95% CI, 3.57–26.96), prior exposure to *T. pallidum* (aOR, 3.49; 95% CI, 1.42–9.01), and undergoing treatment with anxiolytic drugs (aOR, 3.23; 95% CI, 1.21–9.38) were identified as independent risk factors for HBV exposure (Table 3).

**Table 3.** Univariate and Multivariate Logistic Regression Analyses of Risk Factors Associated With HBV Exposure

	Negative (n = 93), No. (%)	Positive (n = 86), No. (%)	Total (n = 179), No. (%)	Crude OR (95% CI)	Multivariate Model, aOR (95% CI)
Sociodemographic characteristics					
MSW	41 (44.09)	21 (24.42)	62 (34.64)	0.4 (0.18–0.83)	…
Age >30 y	43 (46.24)	71 (82.56)	114 (63.69)	4.6 (2.17–10.20)	…
Non-Spanish origin	64 (68.81)	40 (46.51)	104 (58.10)	0.12 (0.01–0.87)	…
Primary education or less	15 (16.13)	33 (38.37)	48 (26.82)	2.82 (1.24–6.76)	…
>5 y of sex work	32 (34.41)	72 (83.72)	104 (58.10)	11.02 (2.17–10.2)	9.35 (3.57–26.96)
Bio-behavioral characteristics					
Condomless sex in the last 12 mo	46 (52.87)	47 (55.29)	93 (54.07)	1.1 (0.6–2.01)	…
Stable partner in the last 12 mo	44 (47.31)	37 (43.02)	81 (45.25)	0.84 (0.46–1.52)	…
Alcohol consumption in the last 12 mo (before or during sex)	69 (74.19)	55 (63.95)	124 (69.27)	0.62 (0.32–1.17)	…
Chemsex in the last 12 mo	23 (25.00)	24 (27.91)	47 (26.40)	1.16 (0.6–2.27)	…
Drug injection (ever in life)	2 (2.15)	3 (3.49)	5 (2.79)	1.64 (0.27–12.72)	…
Drug snorting (ever in life)	73 (78.49)	71 (82.56)	144 (80.45)	1.3 (0.62–2.77)	…
Ever been in prison	8 (8.60)	17 (19.77)	25 (13.97)	2.62 (1.1–6.75)	…
Physical aggression or sexual abuse in the last 12 mo	17 (18.28)	23 (26.74)	40 (22.35)	1.63 (0.81–3.36)	…
Injected steroids in the last 12 mo	5 (5.38)	2 (2.33)	7 (3.91)	0.7 (0.09–3.59)	…
Circumcision	4 (4.40)	16 (19.51)	20 (11.56)	5.27 (1.83–19.07)	…
Anxiolytic treatment in the last 12 mo	15 (16.13)	35 (40.70)	50 (27.93)	3.35 (1.43–8.37)	3.23 (1.21–9.38)
Antidepressant treatment in the last 12 mo	8 (8.60)	17 (20.00)	25 (14.04)	3 (0.96–10.78)	…
Diagnosed of STI in the last 12 mo	20 (21.51)	22 (25.58)	42 (23.46)	1.21 (0.64–2.27)	…
HIV PrEP treatment (ever in life)	3 (3.23)	4 (4.65)	7 (3.91)	1.46 (0.31–7.61)	…
Laboratory testing results					
HCV	1 (1.08)	3 (3.49)	4 (2.23)	3.33 (0.42–67.94)	…
HIV	13 (14.29)	37 (43.53)	50 (28.41)	4.16 (1.73–10.92)	…
CT (any location)^a^	10 (10.99)	5 (6.02)	15 (8.62)	0.52 (0.16–1.53)	…
NG (any location)^a^	11 (12.09)	11 (13.25)	22 (12.64)	1.11 (0.45–2.75)	…
Syphilis (treponemal antibodies)	40 (43.01)	62 (72.09)	102 (56.98)	3.06 (1.44–6.63)	3.49 (1.42–9.01)

Abbreviations: aOR, adjusted odds ratio; CT, *Chlamydia trachomatis*; HPV, human papillomavirus; MSWs, male sex workers; NG, *Neisseria gonorrhea*; PrEP, pre-exposure prophylaxis; OR, odds ratio; STI, sexually transmitted infection.

^a^Positive result in any location (urine, pharyngeal, or anal).

### HBV Vaccination Status and Risk Factors for No Prior Vaccination

As shown in Table 2, concerning immunity conferred by HBV vaccination, 33.7% (91/270) of individuals had postvaccination antibodies (HBsAb) in the absence of infection markers (HBcAb and HBsAg; 19.8% were infected with HIV). Among these, 56.0% (51/91) were unaware of their vaccination status (Table 4).

**Table 4.** Univariate Logistic Regression Analysis of Risk Factors for No Prior HBV Vaccination

	Susceptible^a^ (n = 93), No. (%)	HBV Vaccinated^b^ (n = 91), No. (%)	Total (n = 184), No. (%)	Crude OR (95% CI)
Sociodemographic characteristics				
Age >30 y	43 (46.2)	40 (44.0)	83 (45.1)	0.87 (0.49–1.56)
MSW	41 (44.09)	39 (42.86)	80 (43.48)	1.05 (0.58–1.88)
Non-Spanish origin	91(97.85)	82 (90.11)	173 (94.02)	5.00 (1.25–33.3)
Residency permission	31 (33.33)	36 (40.91)	67 (37.02)	0.72 (0.39–1.31)
Primary education or less	15 (16.13)	18 (19.78)	33 (17.93)	1.28 (0.60–2.77)
Bio-behavioral characteristics				
>5 y of sex work	32 (34.41)	39 (42.86)	71 (38.59)	1.42 (0.78–2.63)
Experienced discrimination in medical facilities (ever in life)	22 (24.18)	24 (27.59)	46 (25.84)	0.80 (0.40–1.56)
Ever been in prison	8 (8.60)	9 (10.11)	17 (9.34)	0.83 (0.30–2.27)
Chemsex in the last 12 mo	23 (25.00)	27 (30.34)	50 (27.62)	0.76 (0.39–1.47)
Drug consumption in the last 12 mo	75 (80.65)	70 (80.46)	145 (80.56)	1.01 (0.48–2.12)
Drug injection (ever in life)	2 (2.15)	2 (2.25)	4 (2.20)	0.95 (0.11–8.33)
Hepatitis B vaccination (self-reported)				
No	22 (23.66)	20 (22.47)	42 (23.08)	…
I don't know	47 (50.54)	31 (34.83)	78 (42.86)	1.36 (0.64–2.94)
Yes	24 (25.81)	38 (42.70)	62 (34.07)	0.57 (0.25–1.26)
HIV	13 (14.29)	18 (20.69)	31 (17.42)	0.63 (0.28–1.38)
Not in HIV PrEP treatment (ever in life)	3 (3.23)	11 (12.36)	14 (7.69)	4.23 (1.27–19.2)

Abbreviations: MSWs, male sex workers; OR, odds ratio; PrEP, pre-exposure prophylaxis.

^a^Negative for HBsAg, HBcAb, and HBsAb.

^b^Positive only for HBsAb.

Among all participants, 34.4% (93/270) tested negative for all serological markers (13.9% were infected with HIV) and were thereby identified as vaccination candidates. However, 25.8% (24/93) of these reported having been vaccinated previously.

Never having received PrEP for HIV (odds ratio [OR], 4.23; 95% CI, 1.27–19.20; *P* = .042) and non-Spanish origin (OR, 5.00; 95% CI, 1.25–33.3; *P*  *=* .043) were associated with no serological evidence of prior vaccination (Table 4).

### Comparison of HBV Prevalence and Presence of Postvaccination Antibodies Between TWSWs and MSWs

HBsAg prevalence was higher in TWSWs than in MSWs, but differences were not statistically significant (2.4% [4/169] vs 0.9% [1/101]; *P* = .42) (Table 2). HBV exposure was significantly higher in TWSWs than in MSWs (38.5% [65/169] vs 20.8% [21/101]; *P* = .002).

The proportion of TWSWs with detectable postvaccination antibodies was significantly lower compared with MSWs (30.8% vs 38.6%; *P* < .001). However, due to a higher proportion of TWSWs with previous infection, the percentage of TWSW candidates for vaccination (30.8%; 52/169) was significantly lower than that of MSWs (40.6% [41/101]; *P* < .001) (Table 2).

## DISCUSSION

Identifying risk factors for HBV infection and describing vaccination status are essential to implementing targeted screening and vaccination strategies within populations exhibiting high-risk behaviors. Although some efforts have been made to address HIV/STIs/viral hepatitis in the female sex worker population, the literature including TWSWs and MSWs remains notably sparse. To the best of our knowledge, we assess for the first time in Spain HBV prevalence and vaccination status in TWSWs and MSWs, which was accomplished at entry in a community-based cohort study in Barcelona.

The overall HBsAg prevalence was 1.9%, 8.4-fold higher than that observed in the general population (0.2%) according to a large seroprevalence study conducted in Spain in 2017–2018, and 3.6-fold higher than the prevalence found in a study conducted among the general population in Catalonia (0.5%) [4, 18]. This prevalence of active HBV infection was somewhat higher than that observed previously in the Sweetie project, conducted between 2017 and 2018 among 147 TWSWs and MSWs in Barcelona (0.8%) [13]. In other studies conducted among TWSWs and MSWs, HBsAg prevalence varied from 1.2% in London [11] to 1.7% in Argentina [19] and 5.2% in the Netherlands [20]. These differences may be attributed to various factors, including gender proportion (TWSWs vs MSWs), differing sex practices, differential access to health services, sample size, and recruitment bias. Therefore, characterization of local HBV epidemiology using diverse strategies for each of these populations is essential to obtaining the reliable data required for designing appropriate prevention and control interventions. Besides recruitment at NGOs, other strategies such as self-sampling at home have been used in our setting in gay, bisexual, and other men who have sex with men and trans women sex workers for HIV, HCV, and other STIs [21, 22], and could also be explored for HBV.

According to the latest CDC recommendations, populations with >1% HBsAg prevalence should be considered a priority group for HBV infection [8], which justifies periodic screening for HBsAg in these SW populations. This recommendation aligns with other guidelines, such as those elaborated by the American Association for the Study of Liver Diseases [23], the European Centre for Disease Prevention and Control [24], and WHO [25], which underscore the importance of community-based screening strategies in hard-to-reach populations.

Regarding HBV exposure, the observed 31.8% prevalence in the present study was 6.0-fold higher than that observed in the general Spanish population (5.3%) [4]. Despite the elevated HBV prevalence, both for HBsAg and HBcAb, observed in the present study, participants with HBV exposure were mainly originally from 4 South American countries that have recorded low to intermediate HBV prevalence since 1990 [1], suggesting that engagement in sex work could be a more significant risk factor for HBV infection than country of origin. In this cohort, engaging in sex work for >5 years was associated with a heightened risk of HBV exposure (aOR, 9.35). This observation aligns with previous studies in the HIV field, where an extended duration of sex work has been associated with infection [26], likely due to cumulative exposure to risk factors. Prior exposure to *T. pallidum* was also associated with risk of HBV exposure (aOR, 3.49), similar to a prior study involving TWSWs and MSWs in Argentina where past or current syphilis (evidenced by positive treponemal antibodies) was a risk factor for HBV exposure [19]. This association was expected due to shared risk factors (such as unprotected sex), and it has been previously described as attributable to the ulcerative lesions induced by this agent, which potentially facilitate easier transmission of HBV [27]. The heightened prevalence of STIs in SW requires a comprehensive approach, integrating preventive and screening measures, and a unified vision to eradicate epidemics [9]. The WHO encourages community-based services for SW, aimed at overcoming structural barriers and expanding service accessibility, ensuring ongoing care in a stigma- and discrimination-free environment [5].

Additionally, this study identified a significant association between the use of anxiolytics and HBV exposure (aOR, 3.23). SWs are often exposed to high levels of trauma, abuse, and stigma, contributing to a higher prevalence of mental health conditions such as anxiety and depression, which may necessitate the use of medications like anxiolytics [22]. The relationship between mental health challenges and increased risk of HBV exposure is complex and multifaceted. These individuals may experience a combination of syndemic conditions, including alcohol use and violence, which can adversely affect their mental state and decision-making abilities [23]. Such conditions can also influence sexual risk behaviors, such as engaging in condomless sex [13], thereby elevating the risk of STI acquisition, including HBV [24]. This association could also reflect that suffering from an infection such as HBV could contribute, together with other underlying factors, to increased anxiety requiring medication.

Notably, 7.4% of the participants were only positive for HBcAb, referred to as “isolated HBcAb positivity.” Individuals with this serological pattern face a higher HBV reactivation risk during immunosuppression and may need a booster vaccination [23]. This pattern often indicates occult HBV infection (detectable HBV DNA in the liver or blood) or past acute HBV infection with loss of HBsAb [28]. The latter is especially common in HIV coinfected patients (10%–45%) [29]. Here, 50% of “isolated HBcAb positivity” cases were coinfected with HIV. However, all participants tested negative for HBV viral load, thus ruling out occult HBV infection. Alternatively, this condition could represent the resolution phase of an acute HBV infection, preceding the appearance of HBsAb [28]. A minority of the “isolated HBcAb positivity” cases might be false positives, given that the false-positive rate of the serological test is 0.002% [30].

While HIV and STI testing and vaccination for hepatitis A and B are recommended in sex workers, many are migrants and experience barriers including stigma when accessing health care services [31]. Additionally, over the COVID-19 pandemic, these barriers may have increased [12]. While 33.7% of SexCohort participants (recruited from mid-2019 to mid-2021) exhibited a pattern compatible with immunity derived from vaccination, 34.4% were negative for all serological markers and were therefore identified as candidates for HBV vaccination. This is in line with a previous study among MSWs conducted in London [11], where the authors found a high prevalence of participants in need for HBV vaccination (61.3%).

Individuals who had never been on PrEP for HIV infection showed a higher risk of not being vaccinated (OR, 4.23). In 2019, the Spanish National Health System started funding PrEP, a strategy using antiretroviral drugs for HIV prevention. In Catalonia, PrEP units conduct screenings for STIs and viral hepatitis (including HBV) during baseline visits and administer HBV vaccination if required [32]. As a result, these services present an excellent opportunity to address other infections such as HBV [33]. Conversely, the proportion of HIV-positive participants with detectable postvaccination antibodies was similar to that observed in HIV-negative participants, which could be related to the fact that a high proportion of participants were migrants, and HBV vaccination practices may vary across countries. In fact, 93% of the cohort hailed from Latin American countries, and non-Spanish origin was linked to a lack of postvaccination antibodies (OR, 5.00). Hepatitis B vaccination has been progressively introduced in these countries: Colombia started in 1994, Venezuela in 2000 with inconsistent coverage over time, Brazil in 1998, and Peru in 1997. Given the cohort's median age of 41, many participants likely missed these vaccination campaigns [34].

Our data underscore a significant lack of awareness regarding HBV vaccination status among these SWs. Specifically, 56.0% of participants with a serological profile indicating presence of postvaccination antibodies denied having been vaccinated. Conversely, 25.8% of participants without any serological markers believed they had been vaccinated. For the latter group, it is also possible that they did not complete the full vaccination schedule, or they might have had low HBsAb titers as HBsAb concentrations can decline over time among those vaccinated, making revaccination advisable [7]. While our study does not delve into the specific causes of vaccination awareness, there is a pressing need to reinforce educational programs on viral hepatitis, complementing them with prevention and screening initiatives for these populations. Catalan initiatives like “Téstate” (a web-based self-sampling strategy for HIV/STIs and HCV detection among MSM) [21] and “Disfruta sin C” (a community-led educational platform promoting HCV diagnosis in MSM practicing chemsex, aiming to reduce its incidence) [35] serve as effective decentralized diagnostic models for at-risk groups. The impact of these initiatives could be amplified by including HBV testing and vaccination.

HBV vaccination, while recommended in sex workers by Catalan [7] and international guidelines [36, 37], needs strengthened interventions, especially delivered at community centers frequented by these populations. The WHO's recent guidelines emphasize the importance of integrated and decentralized testing for infectious diseases like HIV, STIs, and viral hepatitis in community settings, which is crucial to achieve a holistic, people-centered approach [9]. Before HBV vaccination, determining serological status is essential due to the observed high HBV exposure and unawareness in this population. This is consistent with CDC recommendations that consider combined screening and vaccination more cost-effective than just vaccination [8]. Nurse-led outreach strategies have proven to be effective to overcome difficulties in HBV testing and vaccination among female sex workers [38]. However, the requirement of nurses and cold-chain storage for vaccines may represent a drawback for implementation. The HBV-COMSAVA study showcased decentralized community-based HBV care as a viable solution for vulnerable groups like African migrants in Barcelona [39].

In a gender-specific analysis, TWSWs displayed increased HBV exposure compared with MSWs. This elevated exposure among TWSWs might be due to age disparities: 65.9% of TWSWs were over 30, with 68.7% having engaged in sex work for >5 years, whereas only 39.6% of MSWs participants were over 30, with just 27.7% having >5 years in the profession. This suggests greater cumulative exposure in the TWSW group, as previously suggested [40]. The age factor could also imply that many TWSWs might not have benefited from earlier vaccination campaigns, in contrast to the younger MSWs. Intriguingly, while both groups had a high need for vaccination, the requirement was notably higher for MSWs (30.77% in TWSWs and 40.59% in MSWs; *P* < .001). Despite higher vaccination rates in this subpopulation, a lower proportion of previous infection potentially makes these individuals more susceptible to HBV. Therefore, vaccination through specific strategies is paramount for both these vulnerable groups.

Despite the strengths of the present study, there are limitations to note. While the SexCohort project was conducted in 2 community centers in Barcelona, it may not fully represent the entire TWSW and MSW populations of this city. However, it was promoted though several strategies reaching out to SWs through sex work websites, seeking to also involve those who did not frequent community centers. The intended sample size of 300 participants could not be reached, and the low numbers of HBsAg-positive participants found limited the comparison of HBV prevalence between the TWSW and MSW groups. Furthermore, self-reported information on bio-behavioral data, previous HBV testing, diagnosis, treatment, and vaccination might only be somewhat reliable due to limited recall, stigmatization, or poor comprehension. To mitigate this, we ensured private and confidential interviews by trained personnel. Additionally, HBsAb levels can decline postvaccination [41] and even be nondetectable in vaccine nonresponders (5% of immunocompetent individuals and 18%–72% of people with HIV depending on their immune status) [17]; this may have led to an underestimation of vaccinated individuals and an overestimation of those deemed HBV-susceptible, but a second series of HBV vaccination is recommended in nonresponders. Among the limitations, it is noted that tests for the hepatitis delta virus (HDV) were not conducted, as our study was conducted in the context of STI screening in community centers and those testing positive were referred to care for further testing. Especially with the recent approval of bulevirtide for the treatment of hepatitis delta, HDV testing should be performed for the clinical assessment of patients diagnosed with chronic hepatitis B (HBsAg-positive). Lastly, although the SexCohort project had longitudinal STI testing, HBV tests were limited to baseline samples, preventing us from examining temporal patterns and inferring causal relationships for observed HBV risk factors.

In conclusion, the observed results confirm the high risk for HBV infection to which TWSWs and MSWs are subjected, their low vaccination rates, and the lack of knowledge about their own vaccination status. There is an urgent need to reinforce HBV screening and vaccination programs among these populations as integrated services offered at the community centers commonly accessed by them in an environment free of stigma and discrimination.
